# European consensus meeting of ARM-Net members concerning diagnosis and early management of newborns with anorectal malformations

**DOI:** 10.1007/s10151-015-1267-8

**Published:** 2015-01-22

**Authors:** H. J. J. van der Steeg, E. Schmiedeke, P. Bagolan, P. Broens, B. Demirogullari, A. Garcia–Vazquez, S. Grasshoff-Derr, M. Lacher, E. Leva, I. Makedonsky, C. E. J. Sloots, N. Schwarzer, D. Aminoff, M. Schipper, E. Jenetzky, I. A. L. M. van Rooij, S. Giuliani, C. Crétolle, S. Holland Cunz, P. Midrio, I. de Blaauw

**Affiliations:** 1 Department of Surgery-Pediatric Surgery, Amalia Children’s Hospital-Radboudumc, Nijmegen, The Netherlands; 2 Department of Pediatric Surgery and Urology, Centre for Child and Youth Health, Klinikum Bremen-Mitte, Bremen, Germany; 3 Department Medical and Surgical Neonatology, Newborn Surgery Unit, Bambino Gesù Children’s Hospital-Research Institute, Rome, Italy; 4 Department of Pediatric Surgery, University Medical Centre Groningen, Groningen, The Netherlands; 5 Department of Pediatric Surgery, Faculty of Medicine, Gazi University, Ankara, Turkey; 6 Department of Pediatric Surgery, University Hospital 12 de Octubre, Madrid, Spain; 7 Unit of Pediatric Surgery, University Hospital Wurzburg, Wurzburg, Germany; 8 Department of Pediatric Surgery, Hannover Medical School, Hannover, Germany; 9 Department of Pediatric Surgery, Fondazione IRCCS “Ca Grande”-Ospedale Maggiore Policlinico, Milan, Italy; 10 Department of Pediatric Surgery, Children’s Hospital Dnepropetrovsk, Dnepropetrovsk, Ukraine; 11 Department of Pediatric Surgery, Sophia Children’s Hospital-Erasmus Medical Centre Rotterdam, Rotterdam, The Netherlands; 12 German Self-Help Organization for Anorectal Malformations, SoMA, eV, Munich, Germany; 13 AIMAR, Associazione Italiana per le Malformazione Anorettale, Rome, Italy; 14 VA, Dutch Patient Organization for Anorectal Malformations, Huizen, The Netherlands; 15 Division of Clinical Epidemiology and Aging Research, German Cancer Research Center, Heidelberg, Germany; 16 Department of Child and Adolescent Psychiatry, Johannes-Gutenberg-University, Mainz, Germany; 17 Department for Health Evidence, Radboudumc, Nijmegen, The Netherlands; 18 Department of Pediatric Surgery, St George’s Hospital and University, London, UK; 19 Department of Pediatric Surgery, National Reference Center for Rare Disease on Anorectal Malformations and Rare Pelvic Anomalies (MAREP), Necker-Enfants Malades Hospital, APHP, Paris-Descartes University, Paris, France; 20 Department of Pediatric Surgery, University Children’s Hospital, Basel, Switzerland; 21 Department of Pediatric Surgery, University of Padua, Padua, Italy

**Keywords:** Anorectal malformation, ARM-Net, Krickenbeck classification, Preoperative workup

## Abstract

The ARM-Net (anorectal malformation network) consortium held a consensus meeting in which the classification of ARM and preoperative workup were evaluated with the aim of improving monitoring of treatment and outcome. The Krickenbeck classification of ARM and preoperative workup suggested by Levitt and Peña, used as a template, were discussed, and a collaborative consensus was achieved. The Krickenbeck classification is appropriate in describing ARM for clinical use. The preoperative workup was slightly modified. In males with a visible fistula, no cross-table lateral X-ray is needed and an anoplasty or (mini-) posterior sagittal anorectoplasty can directly be performed. In females with a small vestibular fistula (Hegar size <5 mm), a primary repair or colostomy is recommended; the repair may be delayed if the fistula admits a Hegar size >5 mm, and in the meantime, gentle painless dilatations can be performed. In both male and female perineal fistula and either a low birth weight (<2,000 g) or severe associated congenital anomalies, prolonged preoperative painless dilatations might be indicated to decrease perioperative morbidity caused by general anesthesia. The Krickenbeck classification is appropriate in describing ARM for clinical use. Some minor modifications to the preoperative workup by Levitt and Peña have been introduced in order to refine terminology and establish a comprehensive preoperative workup.

## Introduction

Anorectal malformations (ARM) are rare congenital anomalies treated by pediatric surgeons with different levels of experience. This experience is limited by the small number of cases per center. Defining and evaluating the international classification of ARM and preoperative workup is mandatory to evaluate multicenter treatment and outcome data. In the last decade, the classification of the international conference for the development of standards for the treatment of ARM [1] (Krickenbeck classification; Table 1) has successfully filled that need. In addition, the preoperative workup for neonates with an ARM suggested by Levitt and Peña [2] has been adopted throughout the world.

**Table 1:** Krickenbeck classification [1]

Major clinical groups	Rare/regional variants
Perineal (cutaneous) fistula	Pouch colon
Rectourethral fistula	Rectal atresia/stenosis
Prostatic	Rectovaginal fistula
Bulbar	H fistula
Rectovesical fistula	Others
Vestibular fistula	
Cloaca	
No fistula	
Anal stenosis	

The ARM-Net consortium was founded in 2010. It is an international collaboration of pediatric surgeons, geneticists, epidemiologists, and patients’/parents’ organizations [3]. It incorporates 16 participating pediatric surgical centers from eight different countries, together with departments of clinical genetics and/or epidemiology focused on ARM, and three patients’/parents’ organizations (Dutch, German, Italian). The consortium was initiated to increase the knowledge, research, and experience in treating children with ARM. Furthermore, its focus is to develop strategies to initiate and facilitate future multicenter studies on etiology, diagnosis, management, and follow-up.

At a consensus meeting in November 2013, the classification of ARM and the preoperative workup were reviewed. The aim was to evaluate their actuality and appropriateness, thereby improve monitoring of treatment and outcome of these patients.

## Materials and methods

The ARM-Net consortium meeting was held in November 2013 in Nijmegen, The Netherlands. There were 45 participants including 23 pediatric surgeons from nine different countries (France, Germany, Italy, The Netherlands, Spain, Switzerland, Turkey, Ukraine, and United Kingdom). Multiple workshops, all related to the diagnosis and treatment of patients with ARM, were organized. Concerning the classification and preoperative workup, the Krickenbeck classification and the preoperative workup, suggested by Levitt and Peña [2], were used as a template. In our collaborative meeting, participants contributed to a shared proposal to get a consensus that meets the concerns of all members participating in the meeting. At the closure of the workshops, consensus was achieved, and conclusions were summarized.

## Results

### Classification

After its first description of ARM, the Krickenbeck classification [1] has gained overall popularity in the international community of pediatric surgeons. This classification itself seemed a logical sequel to the Wingspread classification elaborated in 1984 [4]. The Krickenbeck classification is clinically oriented, whereas the Wingspread classification was embryologically and anatomically oriented. The ARM-Net meeting agreed that the Krickenbeck classification is the only classification used in clinical practice by all ARM-Net members and appears to be appropriate in describing ARM for clinical use and for comparison of surgical procedures.

### Diagnosis and workup

**Fig. 1:**
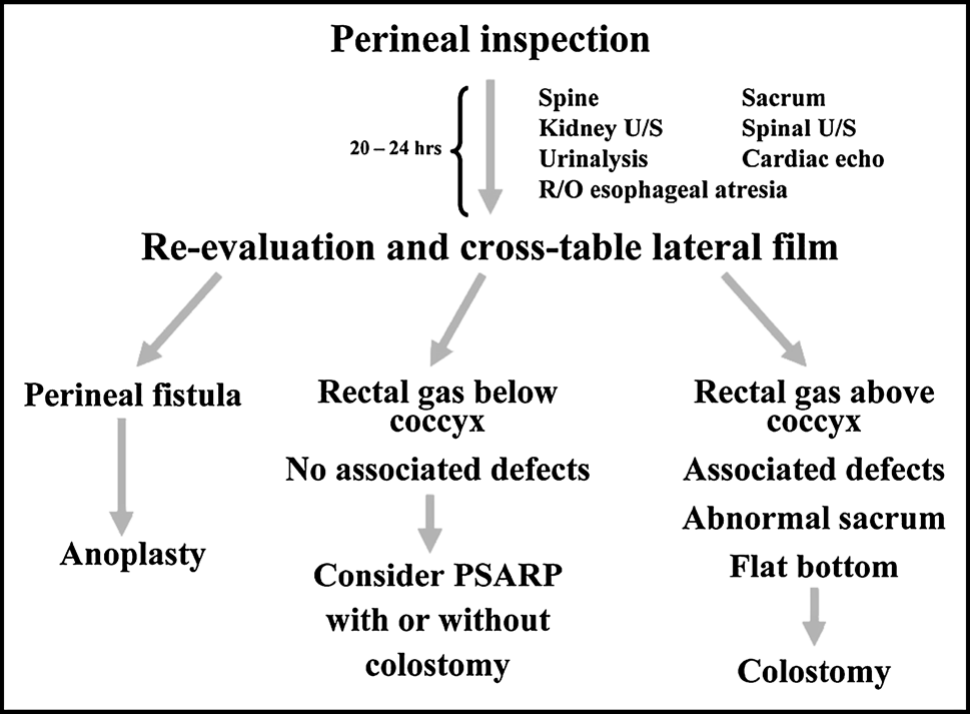
Neonatal workup of male patients as suggested by Levitt and Peña [2]. *PSARP* posterior sagittal anorectoplasty

**Fig. 2:**
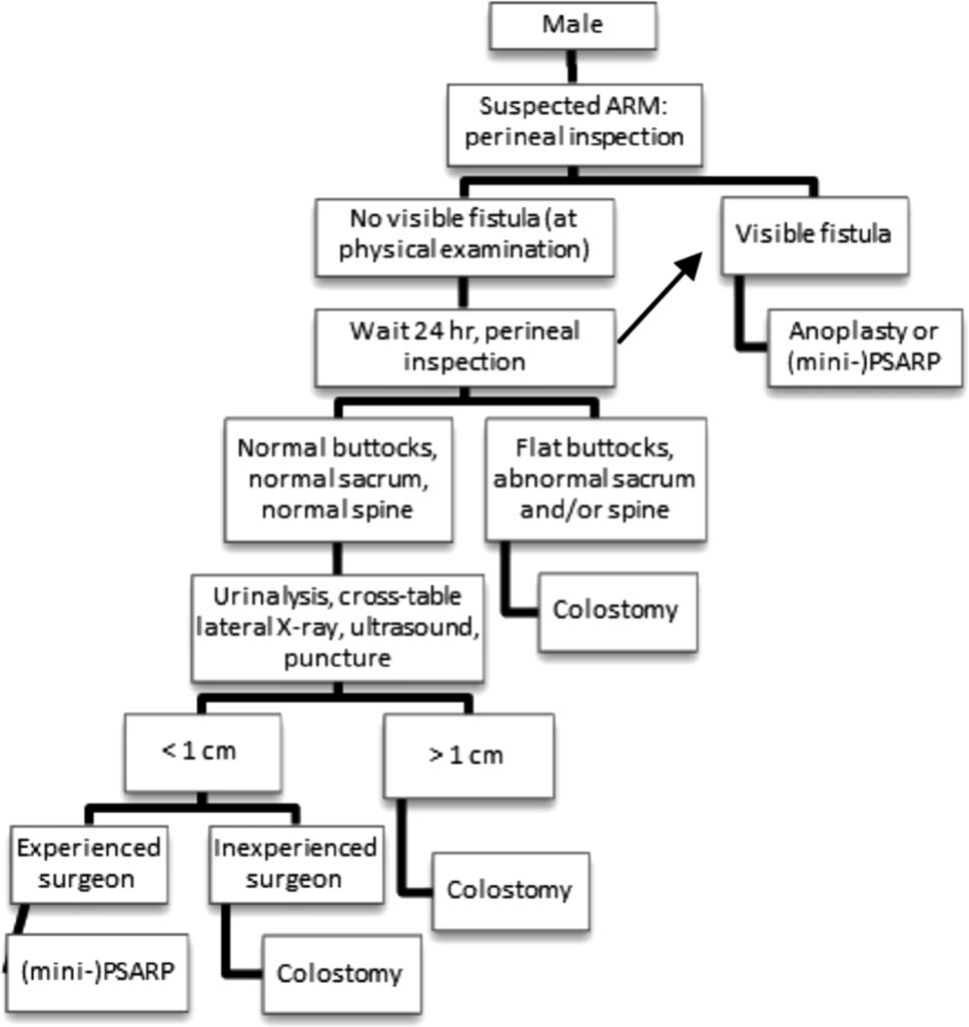
Neonatal workup of male patients suggested by anorectal malformation (ARM)-Net. *PSARP* posterior sagittal anorectoplasty

In Fig. 1, the neonatal workup of the male patient suggested by Levitt and Peña is described, whereas an adapted version by the ARM-Net is displayed in Fig. 2.

In the newborn male with a diagnosis of ARM at the first perineal inspection, screening of the esophagus, heart, kidneys, spinal column, and spine is performed (VACTERL screening). In Levitt and Peña’s flow chart, a re-evaluation and cross-table lateral X-ray are done after 20–24 h.

In the ARM-Net meeting, it was agreed that, in a newborn male with a perineal fistula, a cross-table lateral X-ray is unnecessary, and anoplasty or (mini-) posterior sagittal anorectoplasty (PSARP) can safely be performed (Fig. 2). If there is no visible fistula on physical examination, the patient is re-examined after 24 h to allow time for intraluminal pressure in the rectal pouch to increase, at which point any perineal fistula should develop. This fistula can then be managed with an anoplasty or (mini-) PSARP. In case of no visible fistula, a cross-table lateral X-ray is only indicated in case of normal buttocks, normal spine, normal sacrum, and negative urinalysis on meconium. In case of flat buttocks, abnormal spine, abnormal sacrum, and/or positive urinalysis a (sigmoid) colostomy is made. The cross-table lateral X-ray can be replaced by ultrasound, if an experienced radiologist is available, although the cross-table lateral X-ray remains the first choice imaging examination at the present time. The cross-table lateral X-ray (or ultrasound) in Levitt and Peña’s flow chart is best described as having gas below or above the level of the coccyx (Fig. 1). We choose to measure the lowest level of gas and rectal pouch in centimeters (either by cross-table lateral X-ray or ultrasound) from the overlying skin; in case of a rectum within 1 cm from the covering skin (and as mentioned above with normal buttocks, normal spine, normal sacrum, and normal urinalysis), a primary repair can be performed if an experienced surgeon is available. In all other cases, a colostomy is the treatment of choice. In a few centers, the cross-table lateral X-ray and/or ultrasound can be replaced by a puncture of the skin to determine whether the rectum is within 1 cm distance from the covering skin, but experience with this technique is very scarce, and it should not be performed by unexperienced surgeons.

In conclusion, we agreed on introducing ‘being an experienced surgeon’ as a new variable in performing a primary reconstruction in case of gas less than 1 cm from the covering skin.

**Fig. 3:**
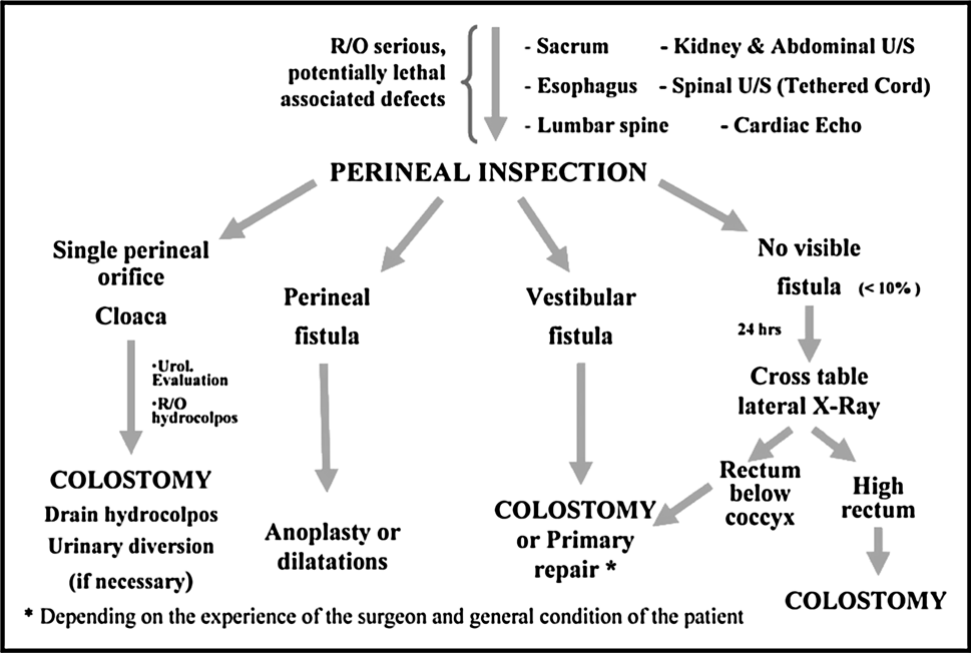
Neonatal workup of female patients as suggested by Levitt and Peña [2]. *US* ultrasound

**Fig. 4:**
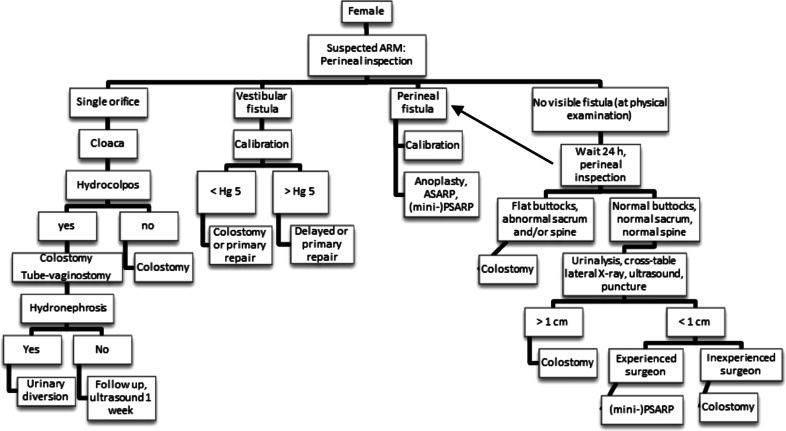
Neonatal workup of female patients suggested by anorectal malformation (ARM)-Net

In females, the ARM-Net flow chart also has some minor modifications compared to Levitt and Peña’s flow chart. The female flow charts are presented in Figs. 3 and 4. Four possible findings can be expected at perineal inspection of a female with an ARM. In case of a single perineal orifice, a persistent cloaca is suspected, and this can present with or without a hydrocolpos; it needs decompression, preferably by tube vaginostomy [5]. The only significant difference in the flow charts is that in the ARM-Net flow chart, a patient with a drained hydrocolpos always needs a follow-up urogenital ultrasound after 1 week. This recommendation is irrespective of an initial hydronephrosis. In case of persistent hydronephrosis despite a drained hydrocolpos, a urinary diversion is recommended.

Patients with a vestibular fistula were a bigger subject of debate. In the Levitt and Peña flow chart (Fig. 3), a colostomy or primary repair is advised. The ARM-Net agreed that a third option, dilatation, is frequently used among ARM-Net members and therefore should be added. Under certain circumstances, the primary repair can be delayed, and a colostomy may be avoided. This protocol may be suitable in a child with low birth weight (<2,000 g) or severe associated congenital anomalies. It might be helpful to decrease perioperative morbidity caused by general anesthesia needed for a colostomy or primary repair. Daily gentle dilatations can be performed by the parents until the scheduled delayed repair. For clarification, a single introduction of a Hegar to estimate the size of the fistula is considered a ‘calibration’. Repetitive introduction of a Hegar as a means of keeping the fistula a certain size, even when increasing the size is not the aim, is considered a ‘dilatation’. A precondition for dilatations should be that they can be carried out painlessly even in the neonate [6]. Members of the ARM-Net have the experience that painful dilatations lead to more inflammation and probably fibrosis, and may cause more constipation in the long term [7]. Additionally, there is a worse outcome regarding future behavioral aspects of continence training at the age of 4–5 years [8, 9], as well as potential psychosocial damage [10]. It was agreed that small vestibular fistulas, without spontaneous bowel movements (usually Hegar size <5 mm), are unsuitable for painless dilatations, and for these cases, a primary repair or colostomy is recommended; again without the need for a cross-table lateral X-ray beforehand.

For perineal fistulas, the same arguments concerning dilatations are valid in case of either a low birth weight (<2,000 g) or severe associated congenital anomalies (Fig. 4).

In case of no visible fistula in females, the same protocol can be used as in males. The cross-table lateral X-ray is only done in case of normal buttocks, normal spine, normal sacrum, and normal urinalysis and with a suspected rectum within 1 cm of the covering skin. An experienced surgeon may perform a primary repair in the neonate.

## Conclusions

The Krickenbeck classification has become the gold standard for the classification of ARMs for most pediatric surgeons of the ARM-Net. Although the preoperative workup of a neonate with an ARM suggested by Levitt and Peña has been the standard of care in the last decade for many pediatric surgeons working with children with ARMs, some minor modifications were suggested by the members of the ARM-Net consortium in order to establish a comprehensive preoperative workup. Defining standards for preoperative management enables different centers and individual pediatric surgeons, geneticists, and epidemiologists to collaborate, to initiate future clinical studies, and compare data on outcome.
